# Spectroscopic and Chromatographic Characterization of Wastewater Organic Matter from a Biological Treatment Plant

**DOI:** 10.3390/s100100254

**Published:** 2009-12-29

**Authors:** Min-Hye Park, Tae-Hwan Lee, Bo-Mi Lee, Jin Hur, Dae-Hee Park

**Affiliations:** 1 Department of Earth and Environmental Sciences, Sejong University, 98 Gunja-dong, Gwangjin-ku, Seoul, 143-747, Korea; E-Mails: p31116@hanmail.net (M.-H.P.); zdrc83@hanmail.net (T.-H.L.); ch20610@hotmail.com (B.-M.L.); 2 Locus Solution Co., Ltd., DMC high-tech center, Seoul, 121-270, Korea; E-Mail: danny@locuss.co.kr

**Keywords:** wastewater, refractory organic matter, specific UV absorbance, fluorescence, molecular weight distribution

## Abstract

Spectroscopic and chromatographic changes in dissolved organic matter (DOM) characteristics of influent and treated sewage were investigated for a wastewater treatment plant (WWTP) with a biological advanced process. Refractory DOM (R-DOM) was defined as the dissolved organic carbon concentrations of the samples after 28-day incubation for this study. Specific UV absorbance (SUVA), hydrophobicity, synchronous fluorescence spectra and molecular weight (MW) distributions were selected as DOM characteristics. The percent distribution of R-DOM for the effluent was much higher than that of the influent, indicating that biodegradable DOM was selectively removed during the process. Comparison of the influent versus the effluent sewage revealed that SUVA, fulvic-like fluorescence (FLF), humic-like fluorescence (HLF), the apparent MW values were enhanced during the treatment. This suggests that more aromatic and humic-like compounds were enriched during the biological process. No significant difference in the DOM characteristics was observed between the original effluent (*i.e*., prior to the incubation) and the influent sewage after the incubation. This result suggests that the major changes in wastewater DOM characteristics occurring during the biological advanced process were similar to those for simple microbial incubation.

## 1. Introduction

Most of the wastewater treatment systems are currently operated by biological processes for the control of organic matter. Although wastewater organic matter is easily quantified by the measurements of chemical oxygen demand (COD) or total organic carbon (TOC) concentrations, the analyses of the organic matter quality may not be simple because of the involvement of biological processes. For example, the effluent from biological wastewater treatment systems contains various complex organic compounds such as residual degradable and refractory influent substrates, substrate intermediates and end products [1]. Since the primary removal mechanism of the biological processes is based on the utilization of biodegradable organic substrates from sewage, the refractory organic matter (R-OM) in the influent sewage may not be easily removed during the processes.

Analyses of OM characteristics in treated sewage have become important because of recent interest in reuse and/or reclamation of wastewater to resolve water shortages. The treated sewage may be used to irrigate agricultural fields where available resources of freshwater are limited or even to supplement water sources for rivers and lakes in regions with serious shortages in surface water. Much attention has been paid to the characteristics of the organic matter present in the treated sewage because of the effects on the further treatments such as membrane processes [2] or the effects on the fate and the toxicity of organic and inorganic pollutants in receiving water [3].

In general, organic matter constituents can be classified broadly into two compartments depending on their biodegradable versus refractory characteristics. Biodegradable OM is generally removed selectively from biological treatment processes while refractory OM remains in treated sewage. The refractory compartment is considered to be composed of humic substance (HS) components and/or very large macromolecules whereas the biodegradable compartment is generally thought to be composed of smaller molecules or larger non-humic DOM constituents (e.g., carbohydrates, proteins) [4]. However, changes in OM characteristics that occur during biological treatment processes may be much more complicated than expected from such a simple division [5]. For example, soluble microbial products may be released through substrate metabolism as well as by biomass decay during biological processes [6]. In addition, certain labile OM constitutes such as carbohydrates and amino acids may exist in HS-bound form [7]. Jarusutthirak and Amy [6] demonstrated that glucose, a simple labile organic substrate, was transformed into uncharacterized macromolecules with a high MW of >10,000 Da in a laboratory-scaled sequential batch reactor.

Characterizing wastewater effluent OM is also important from the standpoint of watershed management because the wastewater effluent flows into rivers and lakes and it finally impacts the surface water quality. For example, Imai *et al*. [8] suggested that increase in R-OM in the Lake Kasumigaura, one of the largest lakes in Japan, were the result of effluent from upstream wastewater treatment plants. For this study, selected spectroscopic and chromatographic analyses including UV-visible and fluorescence spectroscopy and size exclusion chromatography (SEC) were utilized to examine the changes of the wastewater OM during biological advanced treatment processes. Some prior studies have focused on characterizing the spectroscopic properties and the molecular size of effluent DOM [8,9] while little effort has gone to compare the same DOM characteristics of the influent versus the effluent.

## 2. Experimental Section

### 2.1. Sample Collection and Preservation

The influent and the treated sewage samples were collected using 2L sterile polyethylene bottles from a domestic wastewater treatment plant located in the city of Chungju (Korea). The influent sample was raw sewage that has not passed through any of the treatment facilities in the plant. The facility has a capacity of 75,000 m^3^/day and it adopts the B3 (Bio Best Bacillus) process, one of the biological advanced treatment systems developed in Korea. The B3 reactor is a treatment system modifying the existing aerobic oxidation and activated sludge process [10,11]. The treatment system is composed of four chambers and aeration is allowed to decrease successively by chamber [11]. It is designed to effectively remove organics, nitrogen, and phosphorous due to the growth of the Bacillus species. The sewage samples were refrigerated after collection.

### 2.2. Analytical Methods

The sewage samples were first filtered through a 0.1 mm mesh sieve to remove large suspended solids. Biological oxygen demand (BOD) and COD concentrations were determined following standard protocols. The samples were then filtered using a pre-ashed Whatman GF/F filter to separate into dissolved and solid phases.

Concentrations of DOC were determined directly on acidified, air-sparged samples using a Shimadzu V-CPH analyzer. External DOC standards were prepared using potassium hydrogen phthalate. Particulate organic carbon (POC) concentrations were determined with a CHN element analyzer (Flash EA1112). The relative precision of the DOC and POC analyses were <3% and <2%, respectively, based on repeated measurements (*n* = 7). Total organic carbon (TOC) concentrations of the samples were calculated through the summation of DOC and POC concentrations. Absorption spectra were measured at 1-nm increments over a wavelength range of 200–600 nm with a UV-visible spectrophotometer (Evolution 60, Thermo Scientific), using quartz cuvettes with a 1-cm path length.

The fluorescence measurements were done with a luminescence spectrometer (LS-50B, Perkin-Elmer) equipped with a 20 kW xenon arc lamp within 24 hours of returning from the field. Excitation and emission slit widths were adjusted to 10 nm and 10 nm, respectively. Synchronous fluorescence spectra for excitation wavelengths ranging from 250 to 600 nm were recorded using a constant offset (Δλ = 30 nm) between excitation and emission wavelengths. Preliminary studies showed that synchronous fluorescence spectra with the selected offset successfully captured various fluorescence characteristics [12]. To limit second-order Raleigh scattering, a 290-nm cutoff filter was used for the samples. The fluorescence response to a blank solution (Milli-Q water) was subtracted from the spectra of each sample [13,14]. Finally, fluorescence intensities of the samples were normalized to units of quinine sulfate equivalents (QSE) based on fluorescence measured from a series of diluted quinine sulfate dehydrate solutions in 0.05 M sulfuric acid at excitation/emission wavelengths of 350/450 nm [15]. The samples were diluted prior to fluorescence measurements until UV absorbance at 254 nm was below 0.1 to avoid the inner-filter correction [16,17]. They were also acidified to pH 3.0 to avoid the potential interference of metals [18]. Relative precisions of <2% were routinely obtained based on replicated fluorescence measurements (*n* = 7).

Size exclusion chromatography (SEC) was measured using a high performance liquid chromatograph (Waters model 590) with a UV detector (Waters 486 absorbance detector) and a protein-Pak 125 column. The samples were measured at a detection wavelength of 254 nm. Molecular weight standards included sodium polystyrene sulfonate (PSS) (18 K, 8 K, 4.6 K, and 1.8 K nominal MW, Polysciences, Inc.), salicyclic acid (125 Da, HPLC grade, Aldrich), and acetone (58 Da, HPLC grade, Aldrich). The mobile phase was a 0.1 M NaCl solution maintained at pH 6.8 by adding equal amounts (0.002 M) of NaH_2_PO_4_·H_2_O and Na_2_HPO_4_. The apparent values of the weight-average MW (MW_w_) and the number average MW (MWn) for all samples were calculated based on the observed linear relationship between retention time of the highest peak and the log MW of the standards using the equation previously reported [19]. The mobile phase flow rate was maintained at 1 mL/min.

For the estimation of hydrophobicity, the sample was acidified to pH 2.0 with concentrated HCl before passing it through the DAX-8 resin column (Amberlite™, Aldrich Co.) Hydrophobicity (Ho) was defined as the percentage ratio of the DOC concentration of the effluent from the DAX column to that of the influent. For this study, R-OM was quantified by measuring TOC concentrations remaining in samples after 28 days of dark incubation at 20 °C based on the methodology suggested by Servais *et al*. [20].

## 3. Results and Discussion

### 3.1. Changes in Organic Carbon Concentrations, Hydrophobicity and SUVA Values

Concentrations of BOD, COD, DOC and POC in sewage were reduced after the treatment process (Table 1). The ratio of BOD/COD, which represents the biodegradability of sewage, was 0.72 and 0.42 for the influent and the treated sewages, respectively. The lower ratio of the effluent sewage indicates the preferential removal of biodegradable OM through the biological treatment process. The OM removal appears to be more effective for particulate OM compared to dissolved OM. The reduction rates based on DOC and POC were 82% and 92%, respectively. Our results were consistent with Servais *et al*. [20], which was based on a number of sewage samples collected from different types of wastewater treatment systems. Hydrophilic fractions seem to be more abundant in dissolved OM for the treated sewage compared to influent sewage as revealed by the reduction of hydrophobicity from 35% to 27% (Table 1).

Imai *et al*. [8] have reported that hydrophilic acid fractions were most present in the effluent among the DOM fractions which were separated from XAD resin, cation and anion exchange processes. As expected, R-OM distribution was higher for the effluent versus the influent sewage. R-OM was more distributed in particulate OM.

**Figure 1.**
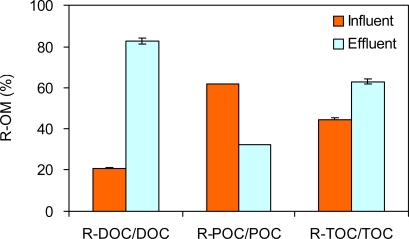
Percent distributions of R-OM for influent and effluent sewage from the wastewater treatment plant.

The R-OM distributions based on DOC (*i.e*., R-DOC/DOC) increased from 21% to 83% during treatment whereas the distributions for the influent and the effluent were 45% and 63%, respectively, based on TOC (Figure 1). This result is not consistent with Servais *et al*. [20], who demonstrated that R-OM was highly distributed in particulate OM for both influent and effluent. Therefore, it appears that the relative difference in the R-OM distributions of dissolved and particulate OM for wastewater is affected by various conditions such as the types of influent and the operational systems. To explain more about this, further investigation using a number of wastewater samples and various treatment operations is required.

The percent hydrophobicity of DOM decreased from 35% to 24% during the biological process (Table 1), indicating that some hydrophobic fractions were presumably removed during the treatment system. In contrast, no reduction of the hydrophobicity was observed for R-DOM. A possible explanation is that some of the biodegradable components may be associated with hydrophobic fractions of DOM. Alternatively, the removal and the generation of hydrophobic DOM components occurred simultaneously while certain fractions produced were resistant to biodegradation. Namour and Muller [21] observed that the hydrophobic distribution increased 40% in the effluent wastewater after 21 days of incubation. They attributed the observation to the fast utilization of hydrophilic components by microorganisms. Hur *et al.* [5] have demonstrated that the hydrophilic biodegradable compounds such as glucose can be transformed into humic-like materials through biodegradation. However, it is likely that microbial transformation of DOM is strongly affected by environmental conditions, the type of DOM, and the associated biological processes [22].

For this study, SUVA values were higher for the effluent versus the influent DOM, indicating that non-aromatic carbon components were preferentially removed by the biological process (Table 2). The enhancement of SUVA values in sewage was also found in prior studies [9,23]. For this study, however, no significant difference in the SUVA value was observed for R-DOM during treatment system (*p* = 0.13).

### 3.2. Changes in Synchronous Fluorescence Spectra

**Figure 2.**
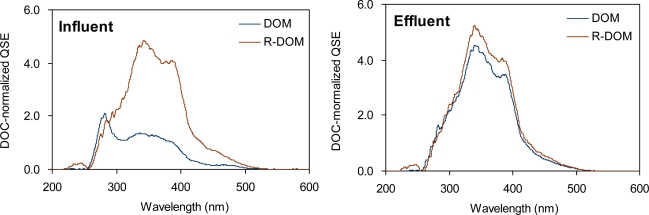
Synchronous fluorescence spectra of influent and effluent sewage from the wastewater treatment plant (DOM: dissolved organic matters, R-DOM: refractory dissolved organic matters).

Fluorescence spectroscopy has been widely used to evaluate the structural and compositional changes of DOM [5,14,16]. Fluorescent organic matter is typically composed of condensed aromatic rings and/or unsaturated aliphatic carbon chains. Synchronous fluorescence spectra of influent and effluent sewages are shown in Figure 2. In general, three peaks could be identified from either of the two samples. The first peak was observed at the wavelength between 250 nm and 300 nm, which was more pronounced for the influent DOM. This peak is associated with the presence of protein- and/or amino acids [12]. The second and the third peaks appeared at the wavelength of ∼350 nm and ∼390 nm, respectively. The former is known to be a predominant fluorescence portion of aquatic DOM and/or aquatic fulvic acid whereas the latter appears to be associated with the humic-like fluorescence, and it is unique for sewage samples [12]. From the synchronous fluorescence spectra, each of the fluorescence characteristics is typically assigned to protein-like fluorescence (PLF), fulvic-like fluorescence (FLF), and humic-like fluorescence (HLF). Recently, Li *et al.* [24] noted a fluorescence peak at a similar location to the HLF that was associated with the accumulation of the extracellular materials produced from biodegradable sewage components during activated sludge process.

For this study, substantial changes in fluorescence characteristics were observed between influent and effluent sewages (Figure 2). The PLF observed for the influent sewage disappeared in effluent whereas the FLF and the HLF characteristics were enhanced after the biological process. Hudson *et al.* [25] also reported that the fluorescence intensity associated with protein/amino acids contained in wastewater declined during a wastewater treatment process. This suggests that amino acids and proteins are used as substrates for microorganisms while fulvic- or humic-like materials may be produced from the biological process. These fluorescence changes were well represented by the ratio of the FLF/PLF increasing from 0.6 to 2.75 during the treatment system (Table 2). Interestingly, the synchronous fluorescence spectrum of the effluent sewage was similar to that of R-DOM in the influent sewage. The similarity between the two DOM implies that the DOM changes that observed during simple microbial incubation may be the same as those occurred during engineered biological advanced process. In fact, there have been many incubation studies reporting the same trends in the fluorescence changes based on other sources of DOM as those observed during the biological process. For example, Hur *et al.* [5] reported the reduction of the PLF and the enhancement of the FLF for leaf litter-extracted DOM during 14 days of incubation.

The difference between the synchronous fluorescence spectra of DOM and R-DOM was much smaller for effluent sewage, which contains an insufficient amount of biodegradable components (*i.e.,* high R-DOM distribution) (Table 2). Therefore, the presence of biodegradable and/or protein-like fluorescent organic materials is likely to be a prerequisite for major fluorescence changes in sewage samples.

### 3.3. Changes in the Molecular Weight Distributions

The apparent MW values of DOM and R-DOM in the influent and effluent sewage is presented in Table 3. The MW_w_ and MW_n_ values were 685 Da and 242 Da for the influent and 849 Da and 501 Da, for the effluent sewage, respectively. Our results agree with Barker and Stuckey [1], who demonstrated that higher MW fractions are distributed in effluent compared to influent sewage. In addition, our apparent MW values for the effluent fall within the MW range reported based on different treatment processes and various types of wastewater [8]. The enhancement in apparent MW values after the biological process may indicate that lower MW fractions were depleted whereas relatively high MW components remained during the process. However, these results may also be explained by the formation of high MW components by microorganisms. For example, Jarusutthirak and Amy [6] showed that high MW components (>10,000 Da) were generated from the utilization of glucose by microorganisms in a sequential batch reactor. The formation of high MW fractions was not limited to the sewage biological treatment system and it seems to be a universal phenomenon for microbial transformation of DOM. The same trend was reported for incubation studies using other sources of DOM such as algal-derived DOM and leaf litter–derived DOM [5,26]. The polydispersity, which is the value of the MW_w_ divided by the MW_n_, represents the heterogeneity of DOM. The lower polydispersity was presented for the effluent compared to the influent sewage, indicating that the effluent has more homogeneous MW distribution. This was probably due to the depletion of the lower MW fractions and/or the enrichment of the high MW components. Similarly for the fluorescence characteristics, no major differences in the apparent MW values were found between R-DOM of the influent and the effluent sewage itself.

**Figure 3.**
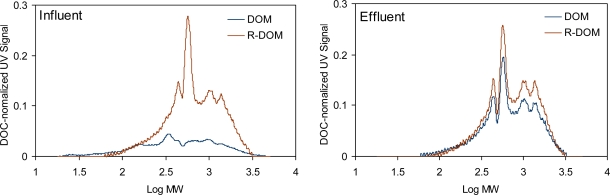
Size exclusion chromatograms of influent and effluent sewage from a wastewater treatment plant (DOM: dissolved organic matters, R-DOM: refractory dissolved organic matters).

For both of the influent and the effluent sewage, MW was broadly distributed from ∼ 100 Da to 3,000 Da (Figure 3). The SEC chromatograms showed that UV-absorbing components were more enriched per organic carbon over a wide range of MW values for effluent (Figure 3). Again, a comparison of the SEC chromatograms for the DOM of the effluent and the R-DOM of the influent sewage revealed that DOM changes by the biological process are the same as those observed for the simple incubation. Four SEC peaks were observed at the MW values of approximately 300 Da, 500 Da, 1,000Da, and 1,500 Da for both samples. The overall pattern of the SEC chromatogram may be influenced by the type of the influent and the condition of the biological operations. [8,6] Nevertheless, the MW values corresponding to the SEC peaks for this study were similar to those reported by Imai *et al*. [8] Imai *et al*. [8] observed that three SEC peaks at the MW values of 330 Da, 640 Da, and 960 Da were present for a number of treated sewage irrespective of the biological treatment operations. Although SEC chromatograms may describe MW distribution of sewage, they are limited to UV-absorbing organic components because of the selectivity of the UV detector.

**Figure 4.**
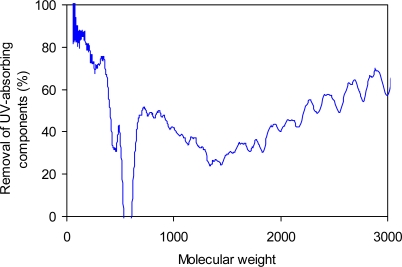
Percentage change in UV signal (at 254 nm) with molecular weight after the biological treatment system.

For more details on the MW distribution changes related to biological treatment system, relative UV removal efficiency was calculated as a function of MW by subtracting UV signal of the effluent DOM from the corresponding initial signal for the influent DOM (Figure 4).

As a result, UV removal efficiency was higher in the relatively low MW range. More than 70% of the UV-absorbing DOM components were removed at the MW value of less than 300 Da whereas the higher MW components were removed to a lesser degree. However, it is difficult to explain the removal rate of UV-absorbing components because it increased with MW at the MW values over 1,500 Da (Figure 4). This unexpected observation may be attributed to the presence of large sized biodegradable DOM components. For example, certain amino acids and carbohydrates may be present as humic-bound form having high MW values. [7] In this case, the partially-labile DOM components might be detected as UV-absorbing species. Chefetz *et al*. [27] have demonstrated using 13-C NMR analyses that high MW and hydrophobic fractions of wastewater samples (*i.e*., humic-like substances) are composed of polymeric structures of covalently linked carbohydrates.

## 4. Conclusions

The DOM SUVA value of domestic sewage increased double after a biological advanced treatment, suggesting that non-aromatic carbon components were selectively removed and aromatic fractions remained during the process. Comparison of the synchronous fluorescence spectra for the influent and effluent sewage revealed that protein-like fluorescence was reduced and fulvic- and humic-like fluorescence was enhanced with the biological system. This indicates that the utilization of labile components is associated with the formation of humic-like materials. The SEC chromatograms showed that low MW fractions were removed through the biological process to a higher degree compared to high MW components. At the MW values of more than 1,500 Da, however, the removal rate increased. This unexpected trend can possibly be explained by the presence of polysaccharides and amino acids in the humic-bound form. The spectroscopic and chromatographic changes in sewage observed during the biological process were similar to those found for simple microbial incubation. The results suggest that common changes in the microbial transformation of sewage DOM are present irrespective of biological processes.

## Figures and Tables

**Table 1.** Organic matter (OM) concentrations and the distribution of hydrophobic fractions for influent and effluent sewage from a wastewater treatment plant.

	**Type**	**BOD (mg/L)**	**COD_Cr_ (mg/L)**	**DOC (mg C/L)**	**POC (mg C/L)**	**TOC (mg C/L)**	**Ho (%)**
**Influent**	OM	109	150	25.1 (0.30)^a^	35.4 (0.08)	60.5 (0.74)	34.8
	R-OM		63.0	5.24 (0.08)	21.9 (0.05)	27.2 (0.41)	64.1
**Effluent**	OM	7.60	16.1	4.50 (0.08)	2.86 (0.01)	7.36 (0.13)	26.9
	R-OM		11.4	3.72 (0.03)	0.92 (0.00)	4.64 (0.04)	65.6

a The standard errors are based on the uncertainties in the precision for each instrument.

**Table 2.** Spectroscopic characteristics of influent and treated sewage from a wastewater treatment plant.

	**Type**	**SUVA**	**PLF/DOC**	**FLF/DOC**	**HLF/DOC**	**FLF/PLF**
**Influent**	DOM	0.98 (0.02)^a^	2.11 (0.03)	1.33 (0.02)	0.96 (0.01)	0.63 (0.01)
	R-DOM^b^	2.10 (0.05)	1.71 (0.03)	4.81 (0.09)	4.05 (0.07)	2.81 (0.05)
**Effluent**	DOM	1.93 (0.05)	1.63 (0.03)	4.49 (0.09)	3.44 (0.07)	2.75 (0.05)
	R-DOM	1.85 (0.04)	1.29 (0.02)	5.11 (0.07)	3.91 (0.05)	3.95 (0.05)

a The numbers in the parentheses are standard errors based on propagating the corresponding value uncertainties.

b R-OM: refractory organic matter (DOC concentrations of the sample remaining after 28-day incubation).

**Table 3.** Weight-average molecular weight (MW_w_), number-average molecular weight (MW_n_) and polydispersity for influent and effluent from the wastewater treatment plant before and after 28-day incubation.

	**Type**	**MW_w_**	**MW_n_**	**Polydispersity**
**Influent**	OM	685	242	2.83
	R-OM^a^	823	504	1.63
**Effluent**	OM	849	501	1.69
	R-OM	871	565	1.54

a R-OM: refractory organic matter (DOC concentrations of the sample remaining after 28-day incubation).
